# Transmembrane protein ATG-9 links presynaptic autophagy with the synaptic vesicle cycle

**DOI:** 10.1080/15548627.2022.2049151

**Published:** 2022-03-29

**Authors:** Sisi Yang, Daniel A. Colón-Ramos

**Affiliations:** a Program in Cellular Neuroscience, Neurodegeneration and Repair, Departments of Neuroscience and of Cell Biology, Yale University School of Medicine, New Haven, CT, USA; b Instituto de Neurobiología José del Castillo, Recinto de Ciencias Médicas, Universidad de Puerto Rico, San Juan, Puerto Rico; c Wu Tsai Institute, Yale University, New Haven, CT, USA

**Keywords:** AP-3, ATG-9, autophagy, clathrin, endocytosis, Golgi apparatus, neuronal activity state, Parkinson disease, synaptic vesicle cycle, synaptojanin 1/UNC-26

## Abstract

Macroautophagy/autophagy occurs preferentially at synapses and responds to increased neuronal activity states. How synaptic autophagy is coupled to the neuronal activity state is largely unknown. Through genetic approaches we find that ATG-9, the only transmembrane protein in the core autophagy pathway, is transported from the *trans*-Golgi network to synapses in *C. elegans* via the AP-3 complex. At synapses ATG-9 undergoes exo-endocytosis in an activity-dependent manner. Mutations that disrupt the endocytosis pathway, including a mutation associated with early onset Parkinsonism (EOP), lead to abnormal ATG-9 accumulation into subsynaptic clathrin-rich foci, and defects in activity-induced synaptic autophagy. We propose that ATG-9 exo-endocytosis links the activity-dependent synaptic vesicle cycle with autophagosome formation at synapses.

Macroautophagy (herein called autophagy) is a cellular degradative pathway essential for neuronal health and homeostasis. In neurites, autophagosome formation increases upon increased neuronal activity state and preferentially occurs near the presynaptic sites – specialized cellular junctions of neuronal communication. Previous studies from our lab demonstrated that the only transmembrane protein in the core autophagy pathway, ATG-9, is transported from the neuronal cell body to presynaptic sites via the synaptic vesicle kinesin, UNC-104/KIF1A. This raised the possibility that ATG-9 localizes to synapses in vesicles to regulate local synaptic autophagy.

**Figure 1.**
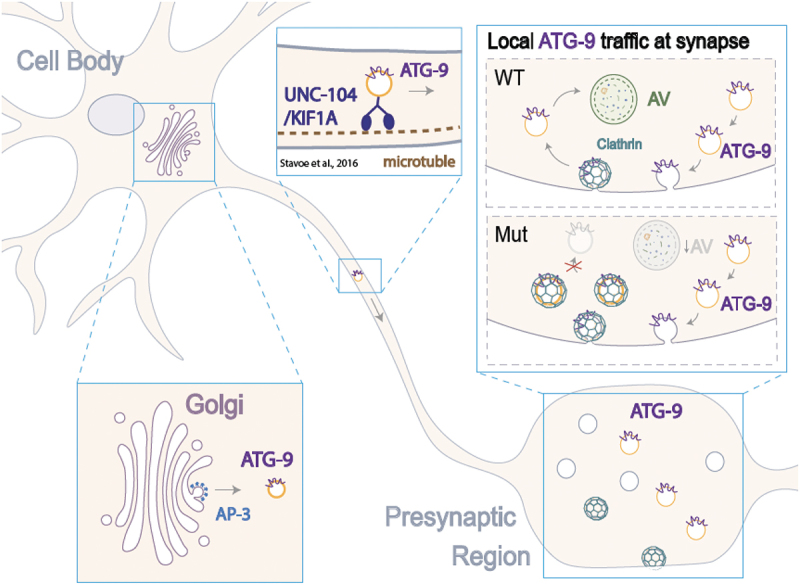
Schematic model of ATG-9 trafficking in *C. elegans* neurons. ATG-9 vesicles are generated from the *trans*-Golgi network via the AP-3 complex (in vertebrates, the primary complex for this function is AP-4, but no AP-4 complex has been identified in *C. elegans*; the role of AP-3 in ATG-9 vesicles in vertebrates is yet to be explored). ATG-9 vesicles transport to the presynaptic region via UNC-104/KIF1A kinesin. At synapses, ATG-9 vesicles undergo exo-endocytosis. Mutations that disrupt endocytosis or an early stage of autophagy result in abnormal ATG-9 accumulation into clathrin-rich synaptic foci, and in defects in activity-induced presynaptic autophagy. The local trafficking mechanisms identified in this study are conserved and similarly act at synapses of *C. elegans* and mammalian nerve terminals. The model is adapted from “Neuronal cells” and “Organelles”, by BioRender.com (2022). Retrieved from https://app.biorender.com/biorender-templates.

To determine whether ATG-9 locates on vesicles at synapses, we performed post-embedding immunogold electron microscopy on transgenic *C. elegans* animals expressing ATG-9::GFP in neurons [1]. We observe that ATG-9 localizes on a subpopulation of vesicles at synapses. We confirmed our EM studies with *in vivo* studies in which we demonstrate colocalization of ATG-9 with synaptic vesicle proteins, RAB-3 and SNG-1/synaptogyrin. Via genetic approaches we then show that in *C. elegans* these vesicles emerge from the Golgi apparatus in an AP-3 dependent manner.

To investigate the role of ATG-9 trafficking at synapses, we performed a candidate genetic screen and find abnormal accumulation of ATG-9::GFP into subsynaptic foci in mutants for genes involved in synaptic vesicle endocytosis, including *unc-26/synaptojanin 1, unc-57/endophilin A* and *dyn-1/dynamin* mutants. Interestingly, the abnormal accumulation of ATG-9 to subsynaptic foci in these mutants can be suppressed in exocytosis mutant *unc-13/Munc-13*, suggesting that ATG-9-containing vesicles undergo exo-endocytosis at synapses. Is the capacity of ATG-9 to undergo exo-endocytosis dependent on synaptic activity? We made use of three conditions that predictably increase or decrease the neuronal activity state of the AIY neurons and find that the ATG-9 mutant phenotype can be predictably manipulated by increasing or decreasing neuronal activity.

To identify the subsynaptic foci onto which ATG-9 abnormally accumulates in the endocytic mutants, we performed *in vivo* cell biological studies in single neurons and determined that ATG-9 accumulates onto clathrin-rich endocytic intermediates in endocytosis mutants. We observe that in a subset of mammalian cultured neurons for endocytic mutants, ATG9A similarly accumulates abnormally into “hot spots” that colocalize with the endocytic protein BIN1/amphiphysin 2. Our findings suggest that in both *C. elegans* and mammalian cultured neurons defective for endocytosis, ATG-9 abnormally accumulates at endocytic intermediates.

Interestingly, mutants that disrupt an early stage of autophagy display similar phenotypes to mutants of synaptic vesicle endocytosis, with ATG-9 also abnormally accumulating into clathrin-rich foci. These observations suggest a link between the autophagy pathway and the synaptic vesicle endocytosis pathway, and support a model whereby ATG-9 in neurons, similar to what has been observed in yeast, can be localized to endocytic structures (we hypothesize, functionally similar to the phagophore assembly site [PAS] in yeast) prior to autophagosome formation.

Is ATG-9 exo-endocytosis important for synaptic autophagy? Consistent with previous findings that synaptic autophagy increases upon increased neuronal activity state, we observe that autophagosome marker puncta LGG-1/Atg8/GABARAP increase upon neuronal stimulation, and that *unc-26/synaptojanin 1* mutants display defects in activity-induced synaptic autophagy. Moreover, specifically engineering, via CRISPR, an allele of ATG-9 lacking a sorting motif linked to endocytosis results in abnormal ATG-9 accumulation to subsynaptic foci and in defects in synaptic autophagy.

*unc-26/synaptojanin 1* is a well-conserved phosphatase, and lesions in its inositol 4-phosphate Sac1 phosphatase domain have been associated with EOP in humans. Given the importance of autophagy in neuronal homeostasis, and the newfound role of *unc-26/synaptojanin 1* in ATG-9 trafficking, we examined whether a similar lesion in *C. elegans* neurons would affect ATG-9 synaptic trafficking. Engineering in *C. elegans*, via CRISPR, the conserved lesion associated with EOP in humans reveals abnormal focal accumulation of ATG-9 in presynaptic nerve terminals in these mutants, as well as defects in neurotransmission and locomotory behavior.

In both yeast and vertebrate cells, Atg9/ATG9 localization is regulated, and this protein underpins autophagosome biogenesis. Our study reveals that in neurons, ATG-9 is actively trafficked to synapses in vesicles that are capable of undergoing the synaptic vesicle cycle in an activity dependent manner, and provides evidence that disruptions of ATG-9 trafficking can affect activity-induced autophagosome biogenesis at synapses (Figure 1). We therefore propose that ATG-9 trafficking could serve as a “sensor” for synaptic activity, linking the activity state of the neuron with autophagosome biogenesis at synapses. In this way ATG-9 could regulate “when and where” autophagosome biogenesis occurs, temporally and spatially “dialing” autophagy to preferentially occur near active synapses, where presumably there will be an increase of damaged synaptic components due to the high activity state. Mechanistically, ATG-9 is a lipid scramblase, and other lipid scramblases are activated upon increases in concentrations of calcium. We speculate that the exo-endocytosis of ATG-9 could serve to activate its scramblase activity, necessary for autophagosome biogenesis. This, or a similar mechanism, would then couple synaptic activity to ATG-9 cycling, the activation of its scramblase domain and autophagosome biogenesis at synapses in response to increased activity state of the neuron.
